# Signal processing in the cochlea: The structure equations

**DOI:** 10.1186/2190-8567-1-5

**Published:** 2011-06-06

**Authors:** Hans Martin Reimann

**Affiliations:** 1 Institute of Mathematics, University of Berne, Sidlerstrasse 5, 3012 Berne, Switzerland

## Abstract

**Background:**

Physical and physiological invariance laws, in particular time invariance and local symmetry, are at the outset of an abstract model. Harmonic analysis and Lie theory are the mathematical prerequisites for its deduction.

**Results:**

The main result is a linear system of partial differential equations (referred to as the structure equations) that describe the result of signal processing in the cochlea. It is formulated for phase and for the logarithm of the amplitude. The changes of these quantities are the essential physiological observables in the description of signal processing in the auditory pathway.

**Conclusions:**

The structure equations display in a quantitative way the subtle balance for processing information on the basis of phase versus amplitude. From a mathematical point of view, the linear system of equations is classified as an inhomogeneous  - equation. In suitable variables the solutions can be represented as the superposition of a particular solution (determined by the system) and a holomorphic function (determined by the incoming signal). In this way, a global picture of signal processing in the cochlea emerges.

## Background

At the outset of this work is the quest to understand signal processing in the cochlea.

### Linearity and scaling

It has been known since 1992 that cochlear signal processing can be described by a wavelet transform (Daubechies 1992 [1], Yang, Wang and Shamma, 1992 [2]). There are two basic principles that lie at the core of this description: Linearity and scaling.

In the cochlea, an incoming acoustical signal *f*(*t*) in the form of a pressure fluctuation (t is the time variable) induces a movement *u*(*x*, *t*) of the basilar membrane at position *x *along the cochlea. At a fixed level of sound intensity, the relation between incoming signal and movement of the basilar membrane is surprisingly linear. However as a whole this process is highly compressive with respect to levels of sound - and thus cannot be linear.

In the present setting this is taken care of by a "quasilinear model". This is a model that depends on parameters, e.g. in the present situation the level of sound intensity. For fixed parameters the model is linear. It is interpreted as a linear approximation to the process at these fixed parameter values. Wavelets give rise to linear transformations. The description of signal processing in the cochlea by wavelet transformations, where the wavelets depend on parameters, is compatible with this approach.

Scaling has its origin in the approximate local scaling symmetry (Zweig 1976 [3], Siebert 1968 [4]) that was revealed in the first experiments (Békésy 1947 [5], Rhode 1971 [6]).

The scaling law can best be formulated with the basilar membrane transfer function . This is the transfer function that is defined from the response of the linear system to pure sounds. To an input signal(1)

i.e. to a pure sound of circular frequency *ω *there corresponds an output *u*(*x*, *t*) at the position *x *along the cochlea that on the basis of linearity has to be of the form(2)

The basilar membrane transfer function is thus a complex valued function of *x *and *ω *> 0. Its modulus  is a measure of amplification and its argument is the phase shift between input and output signals. The experiments of von Békésy [5] showed that the graphs of  and  as functions of the variable *x *are translated against each other by a constant multiple of *log c*. By choosing an appropriate scale on the *x*-axis, the multiple can be taken to be 1. The scaling law is then expressed as(3)

The scaling law will be extended - with some modifications - to include the argument of *ĝ*.

Intimately connected to scaling is the concept of a tonotopic order. It is a central feature in the structure of the auditory pathway. Frequencies of the acoustic signal are associated to places, at first in the cochlea and in the following stages in the various neuronal nuclei. The assignment is monotone, it preserves the order of the frequencies. In the cochlea, to each position *x *along the cochlear duct a circular frequency *σ *= *ξ*(*x*) is assigned. The function *ξ *is the position-frequency map. Its inverse is called the tonotopic axis. At the stand of von Békésy's results, the frequency associated to a position *x *along the cochlea is simply the best frequency (BF), that is the frequency *σ *at which  attains its maximum. The refined concept takes care of the fact that the transfer function and with it the BF changes with the level of sound intensity, at which *ĝ *is determined. The characteristic frequency (CF) is then the low level limit of the best frequency. The position-frequency map *ξ *assigns to the position *x *its CF.

Scaling according to von Békésy's results implies the exponential law(4)

for the position-frequency map. The constant *K *is determined by inserting a special value for *x*.

The scaling law tells us that the function  is actually a function of the "scaling variable"(5)

At the outset of the present investigation it will be assumed that the transfer function *ĝ *is a function of the scaling variable . This is not strictly true, but it simplifies the exposition. In subsequent sections a general theory will be developed that incorporates quite general scaling behavior.

With the availability of advanced experimental data (Rhode 1971[6], Kiang and Moxon 1974 [7], Liberman 1978 [8], 1982 [9], Eldredge et al 1981 [10], Greenwood 1990 [11]), the position-frequency map is now known precisely for many species. Shera 2007 [12] gives the formula(6)

The constant *l *and the "transition frequency" *CF*_1 _vary from species to species. The scaling variable that goes with it is(7)

In the present setting, *x *is the normalized variable (*x *instead of *x*/*l*) and the precise position-frequency map is expressed in the form(8)

*ξ *denotes circular frequency and *K *= *ξ*(0) + *S*. The constant *S *is referred to as the shift.

In the abstract model as it will be developed, much will depend on the definition of the function *σ *that specifies the frequency location. In the present treatment the frequency localization of a function will be defined as an expectation value in the frequency domain.

### Wavelets

The response to a general signal *f*(*t*) with Fourier representation(9)

is given as(10)

Note that the Fourier transform of the real valued signal f satisfies . If the definition of *ĝ *is extended to negative values of *ω *by  then *u*(*x*, *t*) can be written as(11)

The transfer function will be described by a function *h *in the scaling variable:

The response of the cochlea to a general signal *f *can then be expressed as

Setting  and thus *x *= *k *+ *log a *with *k *= *log K*, the scaling function is simply *h*(*aω*). This leads to the equivalent formulation(12)

This is recognized as a wavelet transform. Indeed, with the standard *L*^2^- normalization a wavelet transform *W f *with wavelet *ψ *is defined by

If  is identified with  then(13)

The fact, that the cochlea - in a first approximation - performs a wavelet transform appears in the literature in 1992, both in [1] and in [2].

### Uncertainty principle

The natural symmetry group for signal processing in the cochlea is built on the affine group Γ. It derives from the scaling symmetry in combination with time-invariance. In addition, there is the circle group *S *that is related to phase shifts. Its action commutes with the action of the affine group. The full symmetry group for hearing is thus Γ × *S*. For this group, the uncertainty principle can be formulated. The functions for which equality holds in the uncertainty inequalities are called the extremal functions. They play a special role, similar as in quantum physics the coherent states (the extremals for the Heisenberg uncertainty principle). The starting point in the present work is the tenet that these functions provide an approximation for the cochlear transfer function.

That the extremal functions should play a special role is not a new idea. In signal processing the extremal functions first appeared in Gabor's work (1946) [13] in connection with the Heisenberg uncertainty principle and then in Cohen's paper (1995) [14] in the context of the affine group. In a paper by Irino 1996 [15] the idea is taken up in connection with signal processing in the cochlea. It is further developed by Irino and Patterson [16] in 1997. The presentation in this paper is based on previous work (Reimann, 2009 [17]). The concept pursued is to determine the extremals in the space of real valued signals and to use a setup in the frequency domain, not in the time domain. Different representations of the affine group give different families *E_c _*of extremal functions. The parameter *c *is used to adjust to the sound level and hence to provide linear approximations at different levels to the non-linear behavior of cochlear signal processing.

## Results and Discussion

### Uncertainty principle

This section starts with the specification of the symmetry group Γ × *S *that underlies the hearing process. The basic uncertainty inequalities for this group are then explicitly derived. The analysis builds on previous results (Reimann [17]). A modification is necessary because the treatment of the phase in [17] was not satisfactory. An improvement can be achieved with the inclusion of the term *αĤ *in the uncertainty inequality. This term comes in naturally and it will influence the argument - but not the modulus - of the extremal functions associated to the uncertainty inequalities. It is claimed that the extremal functions derived in this section are a first approximation to the basilar membrane transfer function *ĝ*.

The extremal functions for the basic uncertainty principle are interpreted as the transfer function at high levels of sound. This situation corresponds to the parameter value *c *= 1. With increasing parameter values the extremal functions for the general uncertainty inequality are then taken as approximations to the cochlear response at decreasing levels of sound.

#### The symmetry group

The affine group Γ is the group of affine transformations of the real line **R**. It is generated by the transformation group *τ_b_*(*t*) = *t *+ *b *(*b *∈ **R**) and the dilation group *δ_a_*(*t*) = *at *(*a *∈ **R**, *A *≠ 0). Under the Fourier transform, the action of the dilation group on *L*^2^(**R**, **C **is intertwined to the action of the inverse dilation group . This group also acts directly in frequency space:(14)

The induced unitary action on *L*^2^(**R**, **C**) is(15)

(With this convention, the group action and the induced action are denoted with the same symbol.) Clearly, the invariance property of the basilar membrane transfer function directly reflects this group action.

The action

of the translation group intertwines under the Fourier transform to the unitary action(16)

Of relevance to our considerations is the space *L*^2^(**R**, **R**) of real valued signals of finite energy. Under the Fourier transform it is mapped onto(17)

Both  and  act on . The only action that commutes with both of them is the action(18)

of the circle group S. This is the third distinguished group action. The infinitesimal operators associated with the unitary actions of ,  and  are the skew hermitian operators(19)(20)(21)(22)

The commutator relations are(23)(24)

The operators *Â*,  and *Ĥ *span the Lie algebra of the "hearing group" Γ × *S*, with Γ the affine group and S the circle group.

The basic variables in cochlear signal processing are time t and position × along the cochlea. Clearly  is related to time, whereas *Â *- as will be shown presently - is related to the position.

In our approach the tonotopic axis is given by the exponential law

Under the tonotopic axis dilations  are conjugated to translations (by *log a*) in x:

Here,  is the inverse function with respect to the composition law. The intertwining action

is an isometry in the sense that for all *h *∈ *L*^2^(**R**, **C**)

It intertwines *Â *with :(25)

as the following calculation shows:

The uncertainty principle that goes with the group Γ × *S *can thus been seen as an uncertainty for the determination of time and position.

#### The basic uncertainty inequality

The commutator relation(26)

is at the basis of the uncertainty principle for the affine group. From the inequality

that has to hold for all *κ *∈ **R**, it follows that

In this calculation, the operators *Â *and  can be replaced by  and  respectively. This leads to the new inequality(27)

This inequality is of the same nature as the previous inequality. It can be considered as a more precise inequality, because it holds for all parameter values of *α*, *β *and *ν*. The expression  is minimal for(28)

This *ν *is the decisive parameter. It has the interpretation of an expectation value for the frequency. Later it will be associated with the place along the cochlea.

The uncertainty inequality can thus be stated as(29)

The minimality condition for the parameters *α *and *β *in the expression

is given by the linear system(30)(31)

The coefficients are

In this calculation, the fact that

has been used.

The remaining coefficient is

With  a different meaning can be given to it:

The integrals  and  can be interpreted as expectation values of  in the frequency space and for  in the time space. Roughly, in combination with *Ĥ *operator *Â *controls  and  the time derivative.

We will assume that the parameters *α*, *β *and *ν *are always chosen such that the right hand side in the uncertainty inequality is minimal, i.e. the inequality is formulated in its sharpest form.

The mean deviation from the expectation value *ν *for the modulus of the frequency is(32)

The factor

does not have such a simple interpretation except in the special case *α *= 0. This is treated in [17].

A function h is called extremal, if equality holds for it in the uncertainty relation. The extremal functions are expected to play a special role in the signal processing of the cochlea. In the context of the classical Heisenberg uncertainty relation, the extremal functions are translates of the Gaussian function  under the action of the Heisenberg group. They are called "coherent states". Their significance in signal processing is well established since the appearance of Gabor's work in 1946 [13]. At the outset of the present discussion is however the fact that the cochlea performs a wavelet transform - and not a Fourier transform. The invariance group is Γ × *S *and not the Heisenberg group. It should therefore be expected that the extremal functions as discussed below play the crucial role in the hearing process.

The extremal functions h (in frequency space) satisfy the equation(33)

This is in fact a differential equation:(34)

The solutions are(35)

with real constants *k*, *ε*, *α*, *β*, *κ *and *ν*. Square integrability implies *κ *> 0 and *ν *is the positive frequency expectation value.

From the explicit form it is clear that the space of solutions is invariant under the action of Γ × *S*. The tenet is now:

### The basilar membrane transfer function is given by extremal functions

To be more precise, there exist an extremal function h, normalized by the condition *ν*(*h*) = 1, such that  adequately describes the basilar membrane transfer function *ĝ*:(36)

In this formula, *ξ *= *ξ*(*x*) is the position-frequency map. Note further that

such that(37)

The frequency expectation of *ĝ*(*x*, *ω*) at *x *is thus *ξ*(*x*).

**Figure 1:**
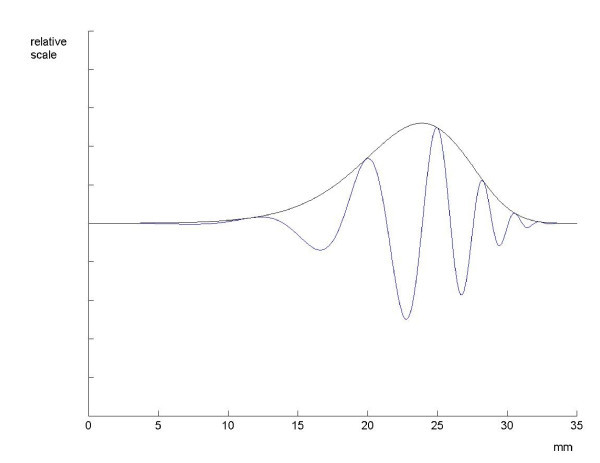
**The extremal function on a relative scale**. The real part and the modulus of the extremal function *h *to the basic uncertainty principle (*c *= 1). In the drawings, the parameters are distance *d *in mm from the stapes  and frequency  in Hz. The extremal function is shown for a fixed frequency *f *as a function of the distance *d *(in mm) to the stapes. The parameters are: *ε *= 2*π*, *α *= -*π*, *β *= 2*π*, *κ *= 4.

The question then arises whether the experiments confirm the tenet. To arrive at a preliminary conclusion, graphs of the modulus and of the real part of the function h are displayed in Figure 1. The parameters are *α *= -*π*, *β *= 2*π *and *κ *= 4.

The classical results by von Békésy (1947) [5] seem to be in favor of such a statement. However the situation is of course not so simple. The basic problem is the non-linearity of the process that associates the movement *u*(*x*, *t*) of the basilar membrane to the input signal *f*(*t*). This process is highly compressive and therefore its description by a transfer function can at best be looked at as an approximation.

Von Békésy's result stem from experiments on dead animals. The outcome can be compared to the experimental results obtained with life animals, yet at high intensities of sound pressure. The above description of the basilar membrane transfer function is therefore taken to be a linear approximation at high levels of sound pressure. In the following section the approach will be modified with the aim of obtaining linear approximations at all levels of sound pressure.

#### General uncertainty inequality for Γ × S

There are various ways that the abstract group Γ × *S *can act on the space . Apart from the natural representation that associates to the corresponding basis elements in the Lie algebra of Γ the operators *Â *and , the general representation considered below is built on the operators  and . The representation of Γ induced by this algebra representation retains the crucial scaling behavior known from the experimental results. It seems to be suitable in the present context, despite the fact that the operator  does not stand for the time derivative any more.

The general representation of Γ × *S *on the space  that will be considered is determined by the representation of its Lie algebra and as such by the operators ,  and *Ĥ*. There is the single non trivial commutator relation:(38)

The uncertainty inequality that goes with it is(39)

At this point it is not clear whether the term  should rather be . In fact the inequality is true for both variants and in both cases, families of inequalities depending on the parameters are obtained. The question is how the extremal functions that are associated to these inequalities vary with the parameters. Yet by choosing the present version one finds that the set of extremal functions is invariant under the action of Γ × *S*. The expression  should been seen as the linear approximation of the skew hermitian operator *Â*. As a result of using both  and , the first as an operator and the latter as an approximation term, this has the effect, that the argument of the extremal function appears in a slightly different context than the modulus.

The extremal functions are obtained from the relation

They satisfy the differential equation(40)

The proportionality factor is . Its choice is arbitrary. All the constants *α*, *β*, and *κ *can in fact be chosen in dependence of the parameter c. This gives a possibility for fine adjustment of the extremal function *h_c _*that describes the linear approximation at level c of the basilar membrane filter.

The solutions of the equation are(41)

with real constants *k*, *ε*, *α*, *β*, *κ *and *ν*. These solutions are in *L*^2 ^if both *κ *> 0 and *ν *> 0.

The parameter in the uncertainty inequality is(42)

This time, the frequency localization of the function h is(43)

and(44)

In accordance with our tenet, the basilar membrane filter is described as(45)

with an extremal function *h_c_*, normalized by the condition *ν*(*h_c_*) = 1.(46)

As before, *ξ *= *ξ*(*x*) is the tonotopic axis. The frequency localization of *ĝ*(*x*, *ω*) as a function of *ω *is *ξ*:(47)

The parameter c allows to express at which level of sound intensity the linearization is specified.

Parameters *c *~ 1 indicate high levels and parameters *c *≫ 1 small levels of intensity.

Experimental results on the basilar membrane transfer function are reviewed in Robles and Rugggero, 2001 [18]. As already pointed out in the previous paper 2009 [17], the shape of the modulus of the transfer function determined at various intensities as given by Rhode and Recio (2000 [19], Figure 1C) is approximately described by the modulus of the extremal functions at the corresponding parameter values. In particular, at a fixed position, the modulus of the transfer function has its peak below the position of the frequency localization and with decreasing intensity of sound level it approaches this position.

Similarly, for fixed *x*, the extremal functions |*ĝ_c_*(*x*, *ω*)| attain their maxima at(48)

With increasing values of *c *this approaches the frequency localization *ξ*(*x*).

With the present setup the argument of the basilar membrane filter is independent of c. The experimental results by Rhode and Recio (2000) show minor changes of phase in dependence of the intensity level. With increasing intensity there is a small phase lag below the characteristic frequency and an equally small phase lead for frequencies above the characteristic frequency. Studies of the impulse response also confirm that the phase is almost invariant under changes in sound level (Recio and Rhode (2000) [20], Shera (2001) [21]). In order to obtain a fine adjustment of the phase data, the parameters *α *and *β *would have to be chosen in dependence of c.

**Figure 2:**
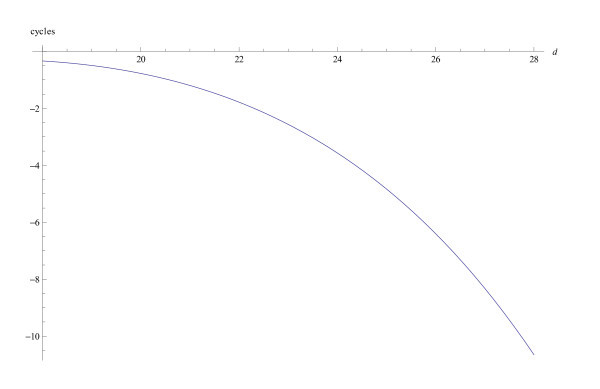
**Phase as a function of *d***. The phase (in cycles) of the extremal function *h *as a function of the distance *d *(in mm) to the stapes. The frequency is 500 Hz. Under the tonotopic axis this value corresponds to *d *= 24.4*mm*. The parameters are: *ε *= 16*π*, *α *= -7.8*π*, *β *= 24*π*.

**Figure 3:**
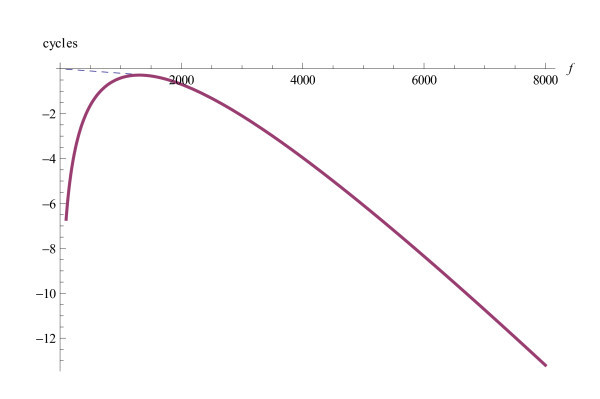
**Phase as a function of *f***. The phase (in cycles) of the extremal function *h *as a function of frequency  at the distance *d *= 10.6*mm *to the stapes. The solid line is the phase as determined by the extremal function. The dashed line is the physiologically correct substitute at low frequencies. Note that the phase values in this region are practically irrelevant for signal processing, because the amplitude values in this region are negligible. Under the tonotopic axis *d *corresponds to the frequency *f *= 4000 Hz. The parameters are the same as in figure 2.

In any case, the phase function has to be a decreasing function both when considered as a function of the frequency *ω *and as a function of the place *x*. The phase of the extremal function does not satisfy this requirement because of the logarithmic term. Yet still, the phase of the extremal function serves as an approximation of the physiological phase function on the interval in which the the absolute value is relevant. At places at which the absolute value is close to zero, the argument is of no significance. In Figure 2 the phase function is pictured for the fixed circular frequency *ω *= 1000 as a function of the distance *d *to the stapes, on the interval that is of physiological relevance. In Figure 3 the phase is pictured as a function of frequency (in Hz). In this figure, the characteristic frequency is 7000*Hz*. The part above about 3000*Hz *is of physiological relevance. The approximation holds in this range. It should be compared with the experimental results by Rhode and Recio (2000 [19] Figure 2E). The part below 3000*Hz *is the mathematical expression for the phase function. It is physiologically not correct, but this is of no significance.

The factor *e*^-*iβω *^in the extremal functions *h_c _*stands for a pure time delay by *β*. Dividing the extremal functions by this factor, one is left with the extremal functions as they would appear if *β *had been set equal to zero in the general uncertainty inequality. They are the extremal functions for the uncertainty inequality(49)

### The structure equations

Extremals for the uncertainty principle satisfy differential equations. Since the membrane transfer function is described by an extremal function and its transforms under the symmetry group and since the extremal functions are preserved under this action, it is possible to derive differential equations for the output of the signal. The resulting equations are called the structure equations.

The derivation starts with the simple case *c *= 1. In this situation differentiation of the wavelet transform *W f*(*a*, *t*) with respect to the parameters of the symmetry group directly leads to a differential equation. In the case *c *> 0 however, the resulting equation is actually a pseudo differential equation. A linearization process for the kernel brings it back to a differential equation that is then satisfied approximately.

The quantities in the equation at first are derivatives of the output function *W f*(*a*, *t*) and its Hilbert transform. A further calculation then shows that the result can be formulated as an inhomogeneous system of linear partial differential equation for the phase and for the logarithm of the amplitude of the output signal. This is particularly satisfying because these are exactly the physiologically relevant quantities.

The point of departure is the tenet that the basilar membrane transfer function is given as

with an extremal function *h_c _*( normalized by the condition *ν*(*h_c_*) = 1).

As mentioned in the section "background" the response to a general signal is interpreted as a wavelet transform:

with

The parameter in the wavelet transform can be normalized such that . The wavelet transform is then(50)

The considerations in this section start from the formula(51)

First the case *c *= 1 is treated. It leads to exact results whereas in the case *c *> 1 an approximation procedure will be applied.

Differentiation with respect to the variable *a *gives

Note that *Â *commutes with . The normalized extremal function h satisfies the differential equation

It follows that

Under the Fourier transform,  is mapped into  and the Hilbert transform H is mapped into *Ĥ*. This gives the basic equation(52)

It is quite remarkable that the differentiation  (this essentially is differentiation with respect to *x*) brings in the Hilbert transform *H*. This transform is a unitary operator on *L*^2^(**R**, **C**). Its square is the negative of the identity operator: *H*^2 ^= -*I*. It extends to a bigger class of functions (to all temperate distributions). On the basic trigonometric functions it operates very simply:

Experimentally, the basilar membrane function is - at least in many studies - determined in terms of the input signals *cos*(*ωt*). It immediately follows that(53)

holds for the functions *f *= *cos*(*ωt*). The linearity assumption then implies that this holds for arbitrary input signals *f*.

Since *Ĥ *commutes with both *Â *and , the basic equation tells us that(54)

The Hilbert transform thus appears naturally in this setting. This is the justification to study the "analytic wavelet transform". Taking the factor  into account this is(55)

The complex valued function *Z f *then satisfies(56)

Notice the shift by  that has its origin in the factor .

The basic equation can now be reformulated as a system of equations in the polar coordinates

The calculation in real terms starts with the observation that on one hand

whereas on the on the other hand

This gives the first equation(57)

Similarly, on one hand

On the other hand

This gives the second equation(58)

The calculation in complex notation makes use of the fact that

The basic equation is then(59)(60)

Dividing by *Z f *it follows immediately that(61)

The case *c *≠ 1 is now being treated in a similar spirit. Recall that the normalized extremal functions (*ν *= 1) satisfy the differential equation

and that the basic equation derives from

The case *c *≠ 1 will not directly lead to a differential operator, because the operator  is not the Fourier transform of a differential operator (unless c is an odd natural number). It is however possible to use a linear approximation for  near the frequency expectation value of *h_c _*i.e. at the point *ω *= 1:(62)(63)

The above equation is then approximated by

With the consequence that(64)

The calculation for the analytic wavelet transform *Z f *= *u *+ *iHu *then proceeds as above. Only the constants are slightly different. In the sequel the notation *γ *= *cκ *will be used. Recall that in prospective refined adjustments the parameters *α*, *β *and *γ *may vary with *c*.

The structure equations are(65)(66)

They combine to the complex equation(67)

Equality holds if *c *= 1.

Under the tonotopic axis *ξ*(*x*) = *Ke*^-*x*^, *x *= *k *+ *log a*, the derivative  transforms into :(68)

The structure equations can be written in *x*, *t*-coordinates:(69)

### Consequences of the structure equations

Signal processing in the cochlea is non-linear. The main - but certainly not the only - source of non-linearity is the compressive nature inherent in the hearing process. In the abstract model pursued here this is taken care of with a single parameter that represents the level of sound intensity. The model then describes the linear approximations at these levels. The structure equations are at the core of this abstract model, in fact they comprise all the essential features. First of all, they are linear (as would be expected from a linear approximation). From a mathematical point of view, the equations therefore are very simple. On top, the system is quite special. With respect to suitable variables it represents an inhomogeneous -equation. Its solutions can be realized in complex form as products of two factors, the first of which is entirely determined by the system and the second is a holomorphic function that can be calculated from the signal. At every level *c *it is thus possible to associate to an input signal in a unique way a holomorphic function that describes the output signal in terms of the physiological parameters.

The phase and the logarithm of the amplitude are used in the description of the experiments and they are omnipresent in all the representations of the auditory pathway. In themselves they are of limited significance, because they are not coded as such. What really is essential in any cochlear or in any neural model are the changes of these quantities, both with respect to time and with respect to the place. The structure equations precisely relate the local and temporal derivatives of phase and (logarithm of) amplitude.

The geometry of the cochlea implicitly is inherent in the extremality property of the basilar membrane filter. But in the structure equations this only shows in terms of the constants. The implicit appearance of the tonotopic axis is an expression of the basic invariance principle that stands at the outset of all considerations.

The structure equations clearly exhibit the dichotomy in cochlear signal processing. The signals can either be analyzed in terms of their phase or in terms of their amplitudes. Assume that there is complete information on phase changes, i.e. the quantities  and  are known. Then the second equation

can be solved for . Inserted in the first equation

this then determines . Conversely, the complete knowledge of amplitude information determines the phase information. From an abstract point of view, phase information and amplitude information each individually contain the full information of the signal. In the auditory pathway both phase and amplitude information is being processed. It is commonly assumed that phase information dominates in the low frequency range and amplitude information in the regions that process high frequencies. The equations tell us that phase processing and amplitude processing are equally significant.

The complex equation

shows that there is also a twofold way of data processing with respect to time and with respect to the place. Complete information on derivatives with respect to the position gives complete information on time derivatives - and vice versa.

The structure equations are so simple that they can be solved in explicit mathematical terms. In its complex form the structure equation is the linear inhomogeneous equation(70)

The general solution of an inhomogeneous linear differential equation can be presented as the linear combination of a particular solution (any chosen solution of the equation) and the general solution of the associated homogeneous differential equation.

A particular solution *log Y_p _*of the above complex equation is the function(71)

Its distinguished feature is the time independence. It follows that the general solution *log Y *is of the form(72)

for some function *X *satisfying the homogeneous equation(73)

This leads to the product representation(74)

(As a side remark, observe that the complex structure equation is obtained from the basic equation(75)

after division by *Z f*. Writing

it is then clear that the homogeneous differential equation for *X *also holds at the zeros of *X*.) With the variable change

the homogeneous equation turns into a - equation for the transformed function(76)

This then shows that the solutions of the linear inhomogeneous equation have the representation(77)

with *G *a holomorphic function in the variable *z *= *t *- *aβ *+ *iaγ*. Since *a *> 0 (and *γ *> 0) it is defined in the upper half space {*z *∈ **C **: *Im z *> 0}. The function *G*(*z*) is uniquely defined up to a constant.

The situation can now be summarized as follows: An incoming signal *f*(*t*) gives rise to a family of analytic wavelet transforms(78)

depending on the parameter *γ *= *cκ*. The functions *Z f *approximately satisfy the complex structure equation. The solutions(79)

of the equation(80)

are then expected to provide approximations for *Z f *(with equality for *c *= 1).

The functions *G *are holomorphic and depend on the parameter *γ*. They can in fact be determined directly from the Fourier transform of the incoming signal *f*(*t*). Since the system is linear, the superposition principle holds:

If *f *= *f*_1 _+ *f*_2 _is the superposition of two incoming signals *f*_1 _and *f*_2 _to which the holomorphic functions *G*_1_(*z*) and *G*_2_(*z*) are associated, then the holomorphic function for *f *is(81)

All that has to be done is to calculate the holomorphic functions that correspond to to the basic functions *f*(*t*) = *Acos*(*νt *+ *ϑ*). In the following section these are identified as the functions(82)

The Fourier representation(83)

then tells us that the holomorphic function associated to *f *is(84)

The conclusion is that the holomorphic functions *G*(*z*) with *z *= *t *- *aβ *+ *iaγ *provide approximate solutions to the structure equation(85)(86)

The relevant expressions in the structure equations can then be calculated from the derivative of *F *(*z*) := *log G*(*z*):(87)(88)(89)(90)

### Examples

#### Pure sounds

For the input signal

the quantities *log r*(*a*, *t*) and *φ*(*a*, *t*) can be calculated explicitly from the formula

This is

and for the derivatives

The first structure equation gives

This is in fact the correct linear approximation. It is equivalent with the linear approximation of |*aν*|*^c ^*at *aν *= 1:

The second structure equation is satisfied as an equality.

From the complex structure equation the holomorphic function associated to *f*(*t*) = *cos νt *can be determined:

With the above approximation this is

(with the abbreviation *cκ *= *γ*). Observe that for *c *> 1

unless *aν *= 1. The approximate value for *log *|*Z f*| therefore is an over estimation. Together with

the result leads to

as the approximate value of *log Z f*.

The associated holomorphic function is thus

with

Note that the holomorphic function *G *associated to the input signal

is(91)

The constants k and *ε *are of little importance and do not show in the structure equations. In the following calculations we set *ke^iε ^*= 1.

#### Amplitude modulation

The amplitude modulated signal

with 0 <*μ *≪ *ν *is described by the holomorphic function

The outcome depends on the ratio between the amplitudes of the coefficients. The frequency *ν *is dominant as long as

With

this gives the estimates

The frequency interval covered is

with

For sufficiently small values of A and *μ *≪ *ν *it includes the entire range along the cochlea that is involved in the processing of the amplitude modulated signal. The function *F *describing this signal in the relevant range can then be estimated by using the approximation *log *(1 + *x*) ≅ *x *for small |*x*|:

and(92)(93)

The result exhibits the basic frequency *ν *as the carrier frequency. But it should be warned that the approximation is valid only in the frequency interval specified above.

The relevant expressions in the structure equation can be calculated from *F'*(*z*):

It can clearly be seen that there is the constant contribution from the carrier frequency and - as the interesting part - a slow oscillation of angular frequency *μ *that stems from *sin *(*μz *+ *const*). Both the amplitude and the phase derivatives show this oscillation.

#### The sound of a violin

**Figure 4:**
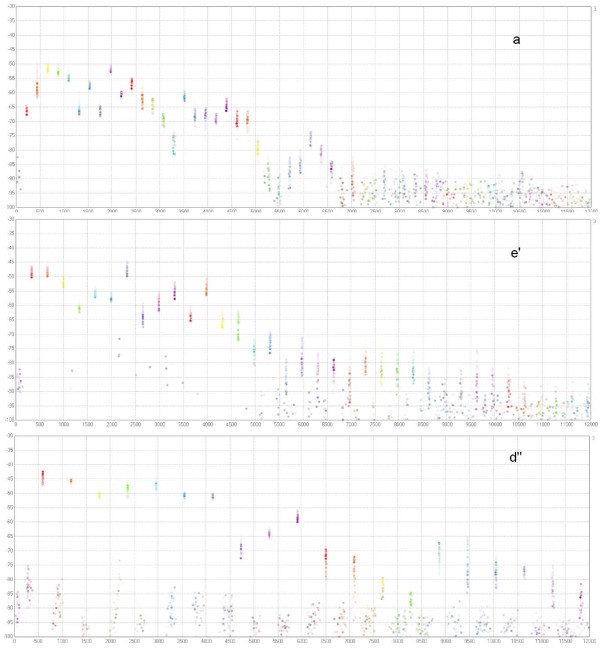
**Spectrograms of three sounds on a new violin**. Spectrograms of the three sounds a, e' and d" on a new violin. The amplitudes of the higher harmonics are determined at short intervals and marked with a point. The intensity of these points is fading with the time. Amplitudes of the harmonics are shown on a relative scale (in dB). More than 20 higher harmonics can be identified. In the first example the level differences of the first 20 harmonics are within a limit of 20 dB.

No doubt, the distinguished feature of a violin sound is the extraordinary big number of harmonics in the frequency spectrum. It is not uncommon to observe around twenty harmonics at an intensity level at which the sounds can still be detected. Except possibly for the first few, the harmonics show a gradual decrease in amplitude with some oscillation. It is conjectured that these properties are in fact characteristic for violins of good quality. Figure 4 shows the amplitudes of the individual harmonics of the violin sounds *a*, *e' *and *d"*. The program "Prisma-Realtime" by Bachmann et al. (2007) [22] uses windowed Fourier transform for this spectrogram. The amplitudes are determined at short intervals and marked with a point. The intensity of these points is fading with the time.

The violin sound has the representation

Figure 4 indicates that |*c_m_*| decreases exponentially in m. In the dB-scale the decrease is roughly linear with slope -2:

(with the approximation 10 ≅ *e*^2.3^).

The associated holomorphic function is

It is a Fourier series  with coefficients

depending on the position (represented by the variable a). The amplitudes

are maximal for

At the place along the cochlea that codes for the angular frequency *nν *i.e. for , the coefficient *d_n _*is thus dominant. In a neighborhood of this *n^th ^*harmonic (near ) the function *F *can be described locally. The calculation exhibits apart from the contribution by the carrier frequency a substantial oscillatory part of angular frequency *ν*.

In a first calculation only the influence of the two closest harmonics is taken into account. The partial signal

corresponds to the function

As before, the approximation

is used. The coefficients

are decomposed as

with *s *= *c*_+ _+ *c*_- _and *d *= *c*_+ _- *c*_-_. This gives the approximation

near the *n^th ^*harmonic. The carrier frequency accounts for the part *inνz *+ *P*(*nν*) + *log A *+ *iθ*. In the structure equation it only participates with time independent terms. But the significant part is the contribution that varies in time with angular frequency *ν *(recall that *z *= *t *- *aβ *+ *iaγ*).

The qualitative picture remains unchanged, if several neighboring harmonics are considered. Near the *n^th ^*harmonic the dominant term in the function *F*(*z*) is expected to be *inνz *+ *P*(*nν*) + *log c_n_*.

This shows the presence of the carrier frequency. The remainder term is approximated by

This function is -periodic in time, with leading term

In conclusion it can be said that locally around  the function *F*(*z*) is of the form(94)

with a well defined remainder term that is -periodic in time. The term *inνz *+ *P*(*nν*) + *log c_n _*shows the presence of the carrier frequency. The relevant information about the violin sound is however contained in the fact that the -periodicity extends over an interval along the cochlea that comprises more than three octaves in the tonal range. The nature of this contribution is similar all along the interval covered by the frequency spectrum of the violin sound. The exceptions are the low harmonics (essentially the first and second ) at which the influence of the neighboring harmonics is very small. Furthermore, the amplitude spectrum of a violin sound very often fails to display monotonicity for the first few harmonics.

With regard to the violins, it should be mentioned that there are considerable differences between different (good quality) instruments. It is believed that the distribution in the first few harmonics very much contributes to the individuality of the violin.

### The impulse response

The response to the impulse function (the dirac function at the origin) is up to rescaling the inverse Fourier transform  of the extremal function

For simplicity it is assumed that *ke*^*iεsgn*(*ω*) ^= 1. Since the Fourier transform of the dirac function is the constant function , the impulse response is(95)(96)(97)

Therefore  is a function of the single variable . This could also be expressed by saying that *tu*(*x*, *t*) only depends on the single variable s - the usual way to formulate the invariance statement.

**Figure 5:**
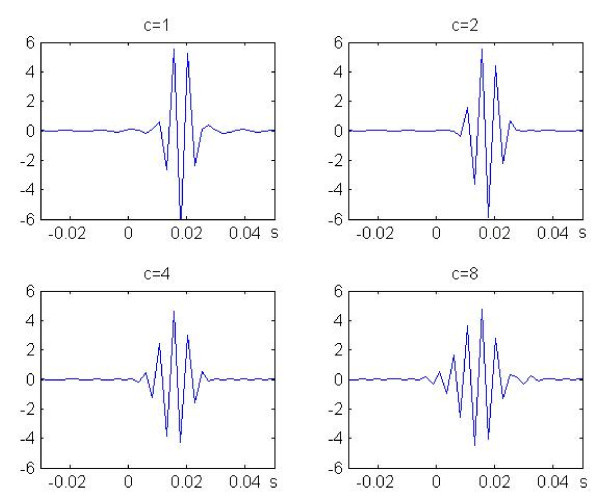
**Impulse response**. The impulse response on a relative scale as a function of time (in seconds), for *κ *= 12 and for various values of *c*. The position along the cochlea corresponds to the frequency 200 Hz.

Figure 5 shows the impulse response for different values of *c*. The impulse response must have its support on the positive half of the time axis. The membrane cannot show a reaction before the impulse arrives. The numerical calculations show that this is almost satisfied. At this point attention should be drawn to a deficiency of the approach. The basic difficulty lies in the concept of using the uncertainty principle. The appropriate thing would be to restrict the class of functions in the uncertainty principle to functions that in the time domain are supported on the positive half axis. However, in the restricted class there are no extremal functions. This can be seen from the fact that the class of extremal functions is translation invariant in the time domain. With the present setting it is not strictly true that the impulse response has its support on the positive half axis. The extremal functions have to be interpreted as a first approximation. They have to be modified slightly such that they really vanish for negative values of t. The numerical calculations show that only small modifications are necessary.

The invariance property of the impulse response mentioned above in combination with the structure equations allow for an explicit approximate calculation of . In the case *c *= 1 this procedure gives the precise value up to a multiplicative constant.

Recall that

was the analytic response. The functions *f *and *g *are then defined starting from *au*(*k *+ *log a*, *t*) as

The derivatives appearing in the structure equations can be expressed in terms of *f*' and *g*':

They are inserted in the structure equations. The result is a system of differential equations for the functions *f' *and *g' *of the variable s.

or equivalently

The complex calculation is quickly done.

All that remains is to determine the integration constant *K*. The inverse Fourier transform  of *h_c _*is then approximated as

This is an exact result if *c *= 1.

The function *log *(*s *- *β *+ *iγ*) is well defined and holomorphic for *Im s *> -*γ*. In this range

can be chosen in the interval (0, *π*). The modulus of the holomorphic function

is then bounded in the upper half plane. Therefore the function *F*(*z*) associated to the impulse response is holomorphic and can be calculated from it. Since *z *and *s *are related by *z *= *t *- *aβ *+ *iaγ *= *a*(*s *- *β *+ *iγ*), it follows that

Therefore(98)

and this function is indeed holomorphic in the upper half plane.

The determination of the integration constant is left open. There is however some information that can be obtained directly from the differential equations for *f *and *g*. The above system is solved for *f' *and *g'*:

From the equation  an estimate for the peak of *log r *at the position given by the angular frequency  can be obtained. The peak is determined by *g'*(*s*) = 0.

This equation tells us how the peak arising from a click travels along the cochlea.

**Figure 6:**
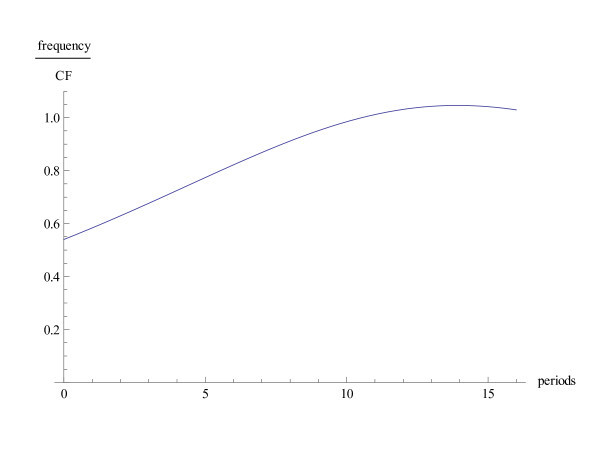
**Frequency glides**. The ratio of momentaneous frequency versus CF as a function of time, measured in periods of CF. The parameters are *α *= -5.3 *π*, *β *= 16 *π *and c = 32 *π*.

Information on the derivative of the phase is obtained from . For this the second derivative of *f *is calculated:

Near the peak of *log r *the second derivative is small and it changes sign between *β *+ *α *and *β *(recall that *α *< 0):

Therefore *f' *is a slightly increasing function at *s *= (*α *+ *β*) and it is slightly decreasing at *s *= *β*. The function *f' *itself takes values close to 1:

The graph of *f'*(*s*) is shown in Figure 6. The parameter values are *α *= -5.3*π*, *β *= 16*π*, furthermore, *γ *= 32*π*.

According to Shera [23] frequency glides in click responses of the basilar membrane have their origin in the dispersion properties of the slow traveling wave. The variable  is the scale invariant variable denoted by 2*πτ *= *t *2*π f_CF _*(*x*) in [23], p.2025. Above, the frequency is expressed in the variable *s *as *φ*(*a*, *t*) = *f *(*s*). This gives

for the instantaneous frequency and hence  for the normalized instantaneous frequency. In [23] p.2025 Shera denotes it by *β_in_*(*τ*) and pictures its graph in figure 2b. The above calculations show that *f'*(*s*) is increasing for values below *α *+ *β*. This can be interpreted as a frequency glide. Note that *f'*(*s*) starts to decrease near *β*. Hence figure 2b in [23] would roughly confirm the present calculations provided that *α *+ *β *≈ 12*π*. There is however a difference in that the present calculation exhibits a dependence of *f' *on the sound level (as represented by *c*) with maximal values for *f' *that are slightly bigger than one.

### General invariance groups

Scale invariance as considered in the previous sections is based on the dilation group *δ_a _*and on the assumption that the tonotopic axis is given by the exponential law *ξ*(*x*) = *K e*^-*x*^. From the experimental data it should however rather be concluded that the symmetry hypotheses are satisfied only locally and in a first approximation. The question therefore arises whether the results subsist qualitatively when these basic assumptions are modified. To answer this question the setup of an abstract model is being presented.

The basic hypothesis is still that the symmetry in cochlear mechanics is given by a one parameter transformation group  acting in phase space. This action can be taken in a quite general form. Ideally it should be possible to adapt it individually to each species. The specific form of the tonotopic axis is to a certain extent independent of the action of the one parameter group. It will be discussed as a separate issue and for the time being the exponential law will be retained.

The transformation group  thus stands at the outset of a general framework for an abstract description of cochlear mechanics. The one parameter group will be enlarged to a bigger group. At first this will be done on the infinitesimal level by defining a multiplier operator  that plays the role of  in the previous sections. Together with the infinitesimal generator  for the one parameter group  and together with *Ĥ*, the action of the Lie algebra of the abstract symmetry group is then completely determined. As an abstract group, the symmetry group is still Γ × *S*, yet the action in phase space will have changed. It will in fact be conjugate to the standard action. The conjugation mapping typically maps a bounded symmetric range in frequency space onto the whole frequency axis. In the application the bounded range will be the interval (-*R*, *R*). The upper bound *R *appears as an absolute frequency bound. In such a model, the inner ear is completely indifferent to signals whose frequency content lies beyond this limit.

As a first issue the wavelet transform for general one parameter groups is being discussed. Next the action of the one parameter group is determined in dependence of the parameter *c *that relates to the overall sound level of the signal. This action will then be extended on the infinitesimal level to all of Γ × *S*. Along with this, the conjugation mapping will be defined.

#### The wavelet transform for general one parameter groups

One parameter transformation groups on **R**^+ ^= (0, ∞) are generated by vector fields *v *: **R**^+ ^→ **R**. Assume that *v *extends to a continuous odd function on **R **and that the solutions *τ_t_*(*ω*) to the differential equation(99)

with initial condition *τ*_0_(*ω*) = *ω *are uniquely determined and depend smoothly on the initial condition *ω*. Then *τ_t _*is a one parameter transformation group of **R**^+^. It extends in an antisymmetric way to all of **R**. At the point *x *= 0 the vector field vanishes and *x *= 0 is a stationary solution of the differential equation. Let us transform the time parameter *t *and set

The one parameter group {*λ_a_*} is then written with a multiplicative parameter *a *∈ **R**^+ ^(the multiplicative group of positive real numbers). It then follows that

Example

The differential equation has the solutions

Assume that the vector field *v *has a finite number of zeroes ±*x_i_*, labeled in ascending order

(the point *x_k _*= ∞ is included though this is not necessarily a zero of *v*). If

then *λ_a_ω *stays in *I_i _*for all *a *∈ R*. If in addition *v *> 0 in *I_i_*, then

and

The variable transform *ζ *= *λ_a_ω*,(100)

gives

whenever the integrals exist. If however *v *< 0 in *I_i _*then the sign changes. Hence in both cases

If *τ_t_*(*ω*) is the solution of the differential equation  with initial condition *τ*_0_(*ω*) = *ω *then the variational equation is

Integrating with respect to *t *gives

This can be written as(101)

The formula expresses that  is the invariant measure for the group action.

The transformation group {*λ_a_*} induces a unitary representation *λ *on *L*^2^(**R**, **C**):(102)

(The same notation *λ_a _*is used for both the group and its unitary representation. To be consistent with the previous notation the group should actually be denoted by , since it will be taken as a group that acts in phase space.)

Note that

is a cocycle for the transformation group {*λ_a_*}.

Given the transformation group {*λ_a_*} in phase space and a function *ψ *∈ *L*^2^(**R**, **C**), define the one parameter family of wavelets by(103)

The wavelet transform with wavelet *ψ *and transformation group {*λ_a_*} is(105)(106)

The isometry properties of the wavelet transform have their origin in the isometry property of the Fourier transform. Applied to *W_ψ _*they give

This equality is integrated with respect to the measure  and the order of integration is interchanged:

With the change of variables *ω *= *λ*_*a*_(*σ*) the integration with respect to  can be transformed into an integration over the interval *I_i _*= (*x_i_*, *x*_*i*+1_) that contains *ω*:

Assume now that *ψ *is a real valued wavelet. Then(108)

From the formula it can be concluded that the full information on *f *is contained in the wavelet transform, provided the integrals are finite and the constants *C_i _*different from zero for all indices. A formal reconstruction can be obtained in terms of the wavelets

(notice the negative sign in the exponent!)

The corresponding wavelet transform is

A similar calculation as before then gives

The function is thus recovered weakly in the sense of *L*^2^-duality by(109)

For the description of cochlear mechanics the reconstruction of the signal from its output at the cochlear level (i.e. from its wavelet transform) is not an issue. No reconstruction is taking place in the auditory pathway. However from the point of information processing it is of relevance to know whether the wavelet transform contains the full information of the original signal.

In the application the wavelet transform will be described by a wavelet with frequency support in [-*R*, *R*]. The above reconstruction process would then give the projection of the signal onto the subspace of band limited signals.

#### Extension of the group action

In the case of the dilation group when *λ_a _*is  and the generating vector field is *v*(*x*) = -*x*, the operator  together with the infinitesimal generator  for the action of the dilation group satisfy the commutator relation

of the Lie algebra of the affine group Γ. For general vector fields *v*, the infinitesimal generator for the group action is(110)(111)

Together with  it will in general fail to span a finite dimensional Lie algebra of operators. However there exists a skew hermitian multiplier operator *M *such that(112)

The multiplier of *M *can be taken in the form(113)

The symmetric real valued function *s*(*ω*) is then determined by the differential equations that results by applying the commutator relation(114)

In any interval void of zeros of the vector field, the function *s *is determined up to a multiplicative factor by

If *v *is a smooth vector field with zeros at ±*x_i _*then *s*(*ω*) will map any interval *I_i _*= (*x*_*i *- 1_, *x*_*i*_) onto the positive real half axis **R**^+^. Furthermore, it will conjugate the action of the transformation group *λ_a _*(restricted to *I_i_*) with the action of the dilation group :(115)

This can be verified by calculating the infinitesimal generator of the conjugate group:

The conjugation map s induces an isometry s* between *L*^2 ^and the subspace  of *L*^2^-functions restricted to -*I_i _*∪ *I_i_*:(116)

In the application, the interval on which *λ_a _*acts will be *I *= (0, *R*) and the vector field will take negative values. The function *sgn*(*ω*)*s*(*ω*) then maps (-*R*, *R*) onto **R **and conjugates *λ_a _*to .

The operators *L *and *M *satisfy the commutator relation of the Lie algebra of the affine group Γ. The action of the group *λ_a _*is thus extended on the infinitesimal level to a Lie algebra action of the affine group.

It can be lifted to Γ = {(*a*, *t*): *a *> 0, *t *∈ **R**} by setting

The circle group action  with infinitesimal generator *Ĥ *remains unchanged and commutes with the Γ - action(117)

The operators corresponding to  are

The only non trivial commutator relation is(118)

For later use note that *λ_a _*commutes with L, whereas

#### The uncertainty inequality

For a given vector field generating the transformation group *λ_a_*, an interval *I_i _*= (*x*_*i *- 1_, *x_i_*) is fixed. The signal space *L*^2^(**R**, **R**) is then restricted to the subspace  of band limited signals with frequencies in -*I_i _*∪ *I_i_*. In this space, the general uncertainty inequality for Γ × *S *can be stated as(119)

with(120)

and

The quantities *ν*(*h*), *α*(*h*) and *β*(*h*) are defined for all functions in  with *Lh *and *M_c_h *in *L*^2^. Under *λ_a _*they transform as(121)(122)(123)

For every parameter *c *> 0 define the frequency localization of h by(124)

Since s conjugates *λ_a _*to , it follows that(125)

Similarly, the phase expectation can be defined by

It satisfies

The equations for the extremal functions is(126)

It has explicit solutions in terms of *s *= *s*(*ω*):(127)

These solutions can either be calculated directly from the differential equation for the extremal solutions or they can be determined from *h_c _*by applying the conjugation mapping s*.

For later purpose note the explicit formula for *λ_a_h_c_*(*ω*). Since *s *conjugates  to *λ_a_*, one has  and therefore(128)(129)

Apart from the factor  this is a function of the single variable *as*(*ω*).

#### Structure equations in the general setting

As in the previous section, assume that *v *is a vector field with zeros at ±*x_i _*that generates a one parameter group *λ_a _*of transformations on -*I *∪ *I *= (-*x_i_*, -*x*_*i*-1_) ∪ (*x*_*i*-1_, *x_i_*). Signals  with frequency content in -*I *∪ *I *can then be analyzed with the group wavelet transform

The Fourier transform  of the wavelet with frequency support in -*I *∪ *I *is taken to be a normalized (with *ν *= 1 ) extremal function for the uncertainty inequality for the group Γ × *S*. The Lie algebra action is determined by the operators *L*, *M_c _*and *Ĥ*. If h is an extremal function with coefficients *α*, *β *and *ν*, then  is also an extremal function. Its coefficients are - *α*, - *β *and *ν*. It is thus possible to take :(130)

The structure equations have their origin in the differential equations(131)

for the extremal functions. In combination with the differentiated form of the wavelet transform

this gives the basic formula(132)(133)

In this situation, a linear approximation for the multiplier operator *M_c _*is inserted. The frequency localization of *λ_a _h_c _*is at *σ *= *σ_c_*(*λ_a _h_c_*). At this position *s^c ^*is approximated by(134)(135)

Note that(136)

with the chosen normalization. Therefore the multiplier operators *M *and *M_c _*- when applied to *λ_a _h_c _*- are approximated by(137)(138)

Altogether, when applied to *λ_a _h_c_*, the expression in brackets in the basic formula above is replaced by

The derivative of the wavelet transform is then expressed in terms of *W f*,  and their Hilbert transforms(139)

(again, the notation *cκ *= γ has been used).

In order to identify the Fourier transform of the wavelet with the cochlear filter, it must be suitably normalized. In case of the dilation group, the normalization  has been used. The function  is localized at . The appropriate normalization for *λ_a _h *is thus . Note that .

As in the section on the structure equation, the analytic wavelet transform of *f *is then defined by(140)

with *σ *= *σ*(*λ_a _h*). The polar coordinates of *Z f *are

The calculation of the general structure equations now proceeds as before. Taken the shift  into account that is caused by the normalizing factor , the coefficients in the above equation are abbreviated by(141)(142)(143)(144)

The general structure equations are(145)(146)

and in their complex form(147)

In the special case that *v*(*x*) = -*x *the coefficients reduce to , *B *= *α*, *C *= -*a β *and *D *= -*aγ*.

The previous structure equations are thus recovered.

The solutions of the complex equation can again be determined. The t-independent particular solution *P*, giving rise to the multiplicative factor *e^P^*, is obtained by solving the equation(148)

If the variable is changed:

then(149)

gives the t-independent factor as a function of the variable *σ*.

Similarly, the homogeneous equation(150)

can be formulated in the variables *σ *and *t*:

The result is the -equation(151)

for the complex variable(152)

Expressed in the variables *σ *and *t*, the solutions of the complex structure equation are thus of the form  with  a holomorphic function in the variable *z*.

#### The abstract models

An abstract model for the cochlea is a model for the cochlea that is based on a one parameter group *λ_a_*. Its cochlear filter *g*(*x*, *ω*) is described by the translates *λ_a _h *of a normalized function *h*. This function is an extremal for the uncertainty relation that goes with the action of the symmetry group Γ × *S *that is determined from the one parameter group *λ_a_*. The frequency localization *σ*(*g*(*x*,.)) will be used in place of the CF. It is independent of the parameter *c *that stands for the level of sound intensity and it satisfies(153)

Assume that the extremal function *λ_a _h *with frequency location *σ*(*λ_a _h*) represents the cochlear filter at x:(154)

Then the point *x*' at which *λ*_*a*' _*h *represents the cochlear filter is given by(155)

The position-frequency map ξ conjugates the group *λ_a _*to the transformation group  along the x-axis. Observe that the generating vector fields *v *and  for the group *λ_a _*and its conjugate  are related by(156)

At the outset of the present studies the group action was given by the dilation group  and the tonotopic axis was defined by(157)

As a result, the group parameter was related to the position by(158)

and the group conjugate to  under the tonotopic axis was the translation group, generated by the vector field .

Basically there are now two different ways in which the tonotopic axis can be built into the abstract model:

1. The starting point is the translation group along the x-axis (generated by the constant vector field). Under the tonotopic axis, this group is conjugated to the group *λ_a _*in phase space. As the prime example, take the experimentally determined tonotopic axis(159)

with "shift" *S *(the inverse to the position-frequency map, see the section "Background").

2. The preassigned group *λ_a _*in phase space is the point of departure. The position-frequency map ξ then conjugates this group to a group  along the x-axis. In general,  will not be the translation group.

Examples for the two variants will be given in the next subsection. Here is an illustration, how the choice of the one parameter group λ_a _can be motivated. There is a graduation of the physiological and geometrical properties along the cochlear duct. The cochlea is arranged in a spiral and the geometric quantities like e.g. the width of the cochlear duct change gradually. The gradation of the physiological data manifests itself e.g. in the change of the elasticity properties of the basilar membrane and in the increase in length of the hair cells and their cilia. The proposition is to take this into account by replacing the translation group along the x-axis (generated by the constant vector field ) by the group  generated by an affine vector field , (*k *constant). This group is then conjugated under the 'rough' position-frequency map *ω *= *ξ*(*x*) = *Ke*^-*x *^to the scaling group *λ_a_*. The generating vector field for this group is then(160)

with(161)

#### Two examples

In this section the procedure of setting up a model according to invariance principles is summarized by explicitly calculating two specific models from the data derived in the previous sections. At the core is the one parameter transformation group *λ_a _*acting in phase space. This action is extended to a group action of the affine group Γ, compatible with the natural action of the circle group *S *in phase space. The general uncertainty principle for Γ × *S *(section 4) leads to families *E_c _*of extremal functions that are invariant under the action of *λ_a_*.

In the cochlea, incoming signals are transformed into neuronal impulses. This process is non linear - in particular with respect to changes in the level of sound pressure of the incoming signal. The parameter *c *stands for an unspecified average level of sound pressure. The action of the cochlea can then be described in its linear approximation at the sound level captured by the parameter *c*. Linear actions are completely determined by the cochlear transfer function *g_c_*(*x*, *ω*).

The models thus obtained will be called abstract models, since they are based on general principles. There are very few parameters in such a model, and these will have to be estimated on the basis of experimental results. Some flexibility lies in the choice of the one parameter group action that stands at the outset of all considerations. Yet the experimental results restrict the choice to group actions that are close to the action of the dilation group. Abstract models vary smoothly in dependence on the one parameter group *λ_a_*. It can be said that the qualitative behavior of abstract models - in particular with respect to the structure equations - is affected very little by the choice of the group *λ_a_*.

In the first example, the underlying one parameter group is the transformation group in phase space generated by the vector field(162)

with flow(163)

that has already been discussed in section 9.1. This vector field is conjugate via the tonotopic axis to an affine vector field along the x-axis. The heuristic motivation for this choice is the gradation of all physical quantities along the cochlea. The affine vector field should take care of this aspect. The infinitesimal generator for the *L*^2^-action is

and the multiplier operator *M *that satisfies [*L*, *M*] = *M *is given by(164)

The symmetric real valued function *s*(*ω*) is determined up to a multiplicative constant by(165)

It maps the interval (-*R*, *R*) onto the real axis. (The zeroes of the vector field are at 0 and at ±*R*. ) The value R appears as an absolute upper bound for the frequency range that is relevant in the hearing process. The extremal functions *h_c _∈ E_c _*for the uncertainty relation have been calculated above (formula 127). For the normalized function (*ν *= 1)(166)

the frequency localization is given by(167)

since the inverse function to *τ *= *s*(*ω*) is(168)

The frequency localization of *λ_a _h_c _*is then(169)

Under the 'rough' position-frequency map *ξ*(*x*) = *Ke*^-*x *^the parameter a is related to the position *x *by the equation(170)

and hence(171)

**Figure 7:**
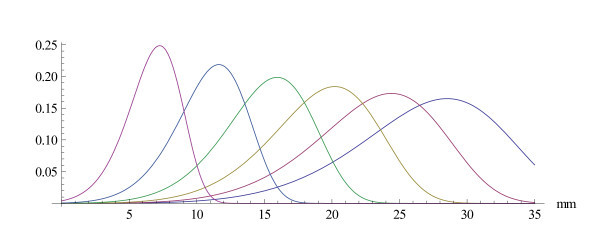
**The cochlear filter for an abstract model**. The amplitude (on a relative scale) of the cochlear filter calculated for the invariance group *λ *generated by the vector field . This is a panoramic view, showing the amplitude on a relative scale as a function of the distance *d *to the stapes for the frequencies *f *= 200, 400, 800, 1600, 3200 and 6400 Hz

**Figure 8:**
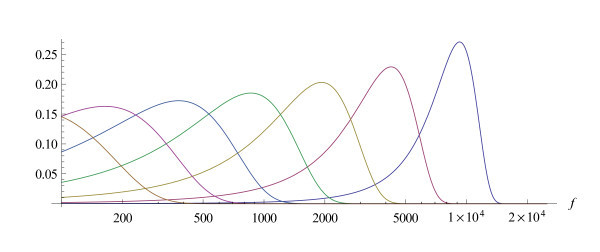
**The cochlear filter as a function of frequency**. The amplitude (on a relative scale) of the same cochlear filter as in the previous figure, but as a function of frequency on a logarithmic scale. The places are taken at distances *d *= 5, 10, 15, 20, 25, 30 and 35*mm *from the stapes.

A parametric view of the modulus of the basilar membrane filter is shown in Figures 7 and 8. The first figure gives it as a function of the distance to the stapes and the second as a function of frequency (on a relative scale). The shape of the amplitude curves changes with CF from wide shallow tunings to sharp tunings at high CF. This is consistent with neuronal tuning curves (Kiang et al. 1965 [24]) and with basilar membrane data (Robles and Ruggero 2001 [18]). Nonlinear analysis techniques that build on Wiener kernels (Temchin et al. 2005 [25], Recio-Spinoso et al. 2005 [26]) or on " zwuis-analysis" (van der Heijden and Joris 2003 [27], 2006 [28]) allow to recover the basilar membrane motion from measurements in the auditory nerve. The resulting panoramic graphs (e.g. [28] Figure 5 or [25] Figure 1 and 2) should be compared with Figures 7 and 8. That the basilar movement in the apical region of the cochlea is difficult to come by with experimental techniques is discussed by Temchin and Ruggero 2010 [29].

The wavelet transform in the cochlea is given by(172)

The function *λ_a _h_c _*is defined at the end of section 9. The expressions for *v *and *s *have to be inserted.

The structure equations are approximate differential equations, which are satisfied by(173)

The equations are

The functions *A*, *B*, *C *and *D *of the parameter *a *are:

The function log *Z f*(*a*, *t*) = *l*og *r*(*a*, *t*) + *i φ *(*a*, *t*) is of the form(174)

with *G*(*z*) a holomorphic function of the variable(175)

The second example is based on the 'physiological' tonotopic axis(176)

(see section "Background") with inverse(177)

The one parameter group *λ_a _*is taken to be conjugate to  under the shift mapping(178)

with shift *S*. Unfortunately, this mapping does not map **R**^+ ^to itself. Hence it has to be modified near *ω *= 0 such that the modified mapping  is monotone, maps **R**^+ ^onto itself and agrees with s for values *ω *≥ *ε*. The positive constant *ε *can be chosen to be arbitrarily small. This difficulty will be suppressed and the notation *s *instead of  will be used for the modified mapping. The vector field for the conjugate one parameter group  is(179)

The frequency localization of the extremal function *λ_a _h *is(180)

The parameter *a *is related to the position by(181)

The normalized function(182)

is thus a function of the "scaling variable"(183)

This is in accordance with the generalized scaling variable defined by Shera (2007, formula 5 p. 2740).

Note that the normalization slightly differs from the normalization  that was used above for the general setting.

**Figure 9:**
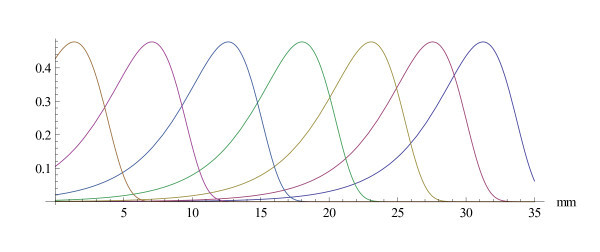
**Adjusting for the position-frequency map**. The amplitude (on a relative scale) of the cochlear filter shown for the physiological position-frequency map  with *K *= 20000, *S *= 200, *l *= 6.6 and parameter *c *= 4. The amplitude is drawn as function of the distance to the stapes for the frequencies *f *= 250, 500, 1000, 2000, 4000, 8000 and 16000 Hz

In Figure 9 the effect of the physiological tonotopic axis is illustrated with a panoramic view of the modulus of the basilar membrane transfer function.

## Conclusions

Signal processing in the cochlea is investigated from an abstract point of view. The time variable *t *and the place variable *x *along the cochlea provide the basic operators  and . These are translated into frequency space. For the place variable the tonotopic axis is used and for the time variable it is the Fourier transform that accomplishes the transfer. The corresponding operators *Â *and  in frequency space do not commute. They satisfy the commutator relation  that displays the relation for the Lie algebra of the affine group Γ. The image under the Fourier transform of the space of acoustic signals is identified as the subspace *L_sym _*of the complex Hilbert space *L*^2^(**R**, **C**). The only one-parameter group of transformations *S *on *L_sym _*that commutes with the action of the affine group is generated by the (Fourier transform *Ĥ *of the) Hilbert transform *H*. The invariance group for the hearing process in the cochlea is thus the product Γ × *S *of the affine group Γ with the circle group *S*.

Signal processing in the cochlea is highly compressive and thus non-linear. The approach pursued here is to fix a parameter *c *for the general level of sound intensity and to specify a linear approximation at this level.

From a mathematical point of view this is done by choosing a different representation of Γ × *S *for every parameter *c*. The non-commutativity of the associated infinitesimal operators gives rise to a family of uncertainty inequalities depending on the parameter *c*. The extremal functions - the coherent states - for these uncertainty relations are expected to play a special role. It is known from the work of Daubechies (1992) and Yang, Wang and Shamma (1992) that the cochlea performs a wavelet transform. The wavelet is defined by the cochlear filter. In the present approach, the linear approximation and with it the cochlear filter depend on the parameter *c*. Comparison with experimental results now suggests that the wavelet that determines the cochlear filter is an extremal function for the uncertainty relation with parameter *c*.

The abstract model as it is derived here from general mathematical concepts has very few parameters and thus gives a very concise picture of signal processing in the cochlea. On the other hand, with just a few parameters at disposition, it will be impossible to capture all the fine structure that has been established experimentally.

With the model at hand, signal processing in the cochlea can be understood in a global way. At the core of the analysis is a system of differential equations - called the structure equations - that hold for the processed signals, i.e. for the wavelet transforms of the acoustic signals. The equations are formulated for physiological observables. In this context these are the derivatives of amplitude and phase. The equations provide us with qualitative and quantitative information on the structure of signal processing in the cochlea. Specifically they give insight into the delicate balance of phase versus amplitude. A global picture emerges since it is possible to present the solutions to the structure equations in terms of holomorphic functions.

As examples, pure sounds, amplitude modulated sounds, clicks and sounds of the violin are subject to special scrutiny. The click response exhibits the wavelet in the time variable (Figure 5). A deficiency of the mathematical model becomes apparent, since the impulse response should have its support in the positive time axis. This would imply that the Fourier transform of the wavelet has a holomorphic continuation to the half space - and this is not the case. In order to remedy this deficiency, the extremals for the uncertainty inequality should be determined within the class of these functions. Yet this class is not compact and such extremals do not exist. However looking at the impulse response, it is clear that the values for *t *< 0 are quite close to zero. The impulse responses at various intensities are thus close to functions with support in the positive time axis.

The analysis of violin sounds tells us that the pitch frequency is present in the movement of the cochlea on a range that covers several octaves.

The symmetries observed experimentally are the most important constituents of the abstract model. On a closer look, these symmetries are only local, that is they hold approximately on bounded time and frequency intervals. The question then arises whether the theory can be adapted to a wider concept of symmetry. In the last section such a theory is developed and it is argued that the principal features of the model can be preserved in this enlarged framework. In particular, the structure equations still remain approximately satisfied. It thus appears that there is some inherent stability in this model.

## Competing interests

The author declares that they have no competing interests.

## References

[B1] Daubechies I Ten lectures on wavelets 1992 Philadelphia: SIAM

[B2] Yang X Wang K Shamma S Auditory representation of acoustic signals IEEE Transaction on Information theory 1992 38 824 839 10.1109/18.119739

[B3] Zweig G Basilar membrane motion Cold Spring Harbor Symposia on Quantit. Biology 1976 40 619 633 10.1101/sqb.1976.040.01.058 820509

[B4] Siebert WM Kolers PA, Eden M Stimulus transformations in the peripheral auditory system Recognizing patterns, MIT, Cambridge 1968 104 133

[B5] von Békéesy G The variation of phase along the basilar membrane with sinusoidal vibrations J Acoust Soc Am 1947 19 452 460 10.1121/1.1916502

[B6] Rhode WS Observations of the vibration of the basilar membrane in squirrel monkeys using the Mössbauer technique J Acoust Soc Am 1971 49 1218 1231 10.1121/1.1912485 4994693

[B7] Kiang NYS Moxon EC Tails of tuning curves of auditory-nerve fibers J Acoust Soc Am 1974 55 620 630 10.1121/1.1914572 4819862

[B8] Liberman MC Auditory-nerve response from cats raised in a low-noise chamber J Acoust Soc Am 1978 63 442 455 670542 10.1121/1.381736

[B9] Liberman MC The cochlear frequency map for the cat: Labeling auditory nerve-fibers of known characteristic frequency J Acoust Soc Am 1982 72 1441 1449 10.1121/1.388677 7175031

[B10] Eldredge DH Miller JD Bohne BA A frequency-position map for the chinchilla cochlea J Acoust Soc Am 1981 69 1091 1095 10.1121/1.385688 7229195

[B11] Greenwood DD A cochlear frequency-position function for several species - 29 years later J Acoust Soc Am 1990 87 2592 2605 10.1121/1.399052 2373794

[B12] Shera CA Laser amplification with a twist: Traveling-wave propagation and gain functions from throughout the cochlea J Acoust Soc Am 2007 122 2738 2758 10.1121/1.2783205 18189566

[B13] Gabor D Theory of communication J IEEE 1946 93 429 457

[B14] Cohen L The scale representation IEEE Transactions on Signal Processing 1993 41 3275 3292 10.1109/78.258073

[B15] Irino T An optimal auditory filter Proc. IEEE Signal Processing Society. Workshop on applications of signal processing to audio and acoustics, New Paltz, NY 1995

[B16] Irino T Patterson RD A time-domain, level-dependent auditory filter: The gammachirp J Acoust Soc Am 1997 101 412 419 10.1121/1.417975

[B17] Reimann HM Uncertainty principles for the affine group Functiones et Approximatio 2009 40 45 67

[B18] Robles L Ruggero MA Mechanics of the mammalian cochlea Physiol Rev 2001 81 1305 1352 11427697 10.1152/physrev.2001.81.3.1305 PMC3590856

[B19] Rhode WS Recio A Study of mechanical motions in the basal region of the chinchilla cochlea J Acoust Soc Am 2000 107 317 3332 10.1121/1.429404 10875377

[B20] Recio A Rhode WS Basilar membrane response to broadband stimuli J Acoust Soc Am 2000 108 2281 2298 10.1121/1.1318898 11108369

[B21] Shera CA Intensity-invariance of fine time structure in basilar-membrane click responses: Implications for cochlear mechanics J Acoust Soc Am 2001 110 332 348 10.1121/1.1378349 11508959

[B22] Prisma-Realtime http://www.prisma-music.ch

[B23] Shera CA Frequency glides in click responses of the basilar membrane and auditory nerve: Their scaling behavior and origin in travelling-wave dispersion J Acoust Soc Am 2001 109 2023 2034 10.1121/1.1366372 11386555

[B24] Kiang NYS Watanabe T Thomas EC Clark LF Discharge patterns of single fibers in the cat's auditory nerve 1965 Cambridge: MIT

[B25] Temchin A Recio-Spinoso A van Dijk P Ruggero M Wiener kernels of Chinchilla auditory-nerv fibers: Verification using responses to tones, clicks, and noise and comparision with basilar-membrane vibrations J Neurophysiol 2005 93 3635 3648 10.1152/jn.00885.2004 15659530 PMC1876724

[B26] Recio-Spinoso A Temchin A van Dijk P Fan YH Ruggero M Wiener-kernel analysis of responses to noise of chinchilla auditory-nerve fibers J Neurophysiol 2005 93 3615 3634 10.1152/jn.00882.2004 15659532

[B27] van der Heijden M Joris PX Cochlear phase and amplitude retrieved from auditory nerve at arbitrary frequencies J Neurosci 2003 23 27 9194 9198 14534253 10.1523/JNEUROSCI.23-27-09194.2003 PMC6740821

[B28] van der Heijden M Joris PX Panoramic measurements of the apex of the cochlea J Neurosci 2006 26 44 11462 11473 10.1523/JNEUROSCI.1882-06.2006 17079676 PMC6674524

[B29] Temchin A Ruggero M Phase-locked responses to tones of chinchilla auditory nerve fibers: Implications for apical cochlear mechanics JARO 2010 11 297 318 10.1007/s10162-009-0197-4 19921334 PMC2862913

